# Vaginal microbiota and the potential of *Lactobacillus* derivatives in maintaining vaginal health

**DOI:** 10.1186/s12934-020-01464-4

**Published:** 2020-11-07

**Authors:** Wallace Jeng Yang Chee, Shu Yih Chew, Leslie Thian Lung Than

**Affiliations:** Department of Medical Microbiology, Faculty of Medicine and Health Sciences, Universiti Putra Malaysia, 43400 Serdang, Selangor Malaysia

**Keywords:** Vaginal microbiota, Vaginal ecosystem, Probiotic, *Lactobacillus*, *Lactobacillus* derivatives, Surface-active molecules

## Abstract

Human vagina is colonised by a diverse array of microorganisms that make up the normal microbiota and mycobiota. *Lactobacillus* is the most frequently isolated microorganism from the healthy human vagina, this includes *Lactobacillus crispatus*, *Lactobacillus gasseri*, *Lactobacillus iners*, and *Lactobacillus jensenii*. These vaginal lactobacilli have been touted to prevent invasion of pathogens by keeping their population in check. However, the disruption of vaginal ecosystem contributes to the overgrowth of pathogens which causes complicated vaginal infections such as bacterial vaginosis (BV), sexually transmitted infections (STIs), and vulvovaginal candidiasis (VVC). Predisposing factors such as menses, pregnancy, sexual practice, uncontrolled usage of antibiotics, and vaginal douching can alter the microbial community. Therefore, the composition of vaginal microbiota serves an important role in determining vagina health. Owing to their Generally Recognised as Safe (GRAS) status, lactobacilli have been widely utilised as one of the alternatives besides conventional antimicrobial treatment against vaginal pathogens for the prevention of chronic vaginitis and the restoration of vaginal ecosystem. In addition, the effectiveness of *Lactobacillus* as prophylaxis has also been well-founded in long-term administration. This review aimed to highlight the beneficial effects of lactobacilli derivatives (i.e. surface-active molecules) with anti-biofilm, antioxidant, pathogen-inhibition, and immunomodulation activities in developing remedies for vaginal infections. We also discuss the current challenges in the implementation of the use of lactobacilli derivatives in promotion of human health. In the current review, we intend to provide insights for the development of lactobacilli derivatives as a complementary or alternative medicine to conventional probiotic therapy in vaginal health.

## Background

Human Microbiome Project (HMP) and Integrative HMP (iHMP) were funded by the National Institutes of Health (NIH). They are interdisciplinary effort that engaged in human microbiome profiling for gut, vaginal, oral, and skin communities [1, 2]. Both projects aimed to unravel the characteristics, distributions, and metagenomics of microbes from those anatomical sites [3]. The findings from HMP are deemed significant to establish the relationship between microbiota changes and pathogenesis of disease, as well as to identify the biomarkers for diagnostic purpose [4].

Human vaginal microbiota comprises a diverse array of beneficial microbes and opportunistic pathogens that inhabit the vaginal milieu [5, 6]. In order to understand the microbiota within human vagina, multiple approaches involving “-omics” technologies have been developed. Molecular approaches that are commonly employed to study the microbial communities are polymerase chain reaction-denaturing gradient gel electrophoresis (PCR-DGGE), DNA pyrosequencing, fluorescence insitu hybridisation (FISH), quantitative PCR, and microarrays [Reviewed in [7]]. Besides, other modern “-omics” technologies such as metabolomics, metagenomics, metatranscriptomics, and proteomics have begun to reinvigorate research into the discovery of functional activity in the microbial communities [8]. The integration of modern multi’omic data is able to decipher the functional insights from complex microbial comuunities through the association of microbial and metabolic profiles with the role in mediating human health [8]. To date, the vast majority of the human microbiota studies utilised 16S rRNA gene sequencing in the identification of complex microbial communities due to its feasibility in inferring the representation of certain microbial communities that cause diseases [9]. Since the advent of technological advances in assessing human microbial diversity, Ravel et al. [10] have successfully identified five distinct bacterial communities by using advanced high-throughput sequencing technology. The indigenous microbiota in the vaginal milieu is believed to be in a symbiotic relationship with the host [11]. Fungi, especially *Candida* species are likely to exist as commensals in the mucous layer of vagina and they form part of the complex vaginal ecosystem with other bacteria [12, 13]. It is suggested that the fluctuation of microbiota and mycobiota composition in women of reproductive age contributed to the temporal dynamics in vaginal communities [11]. In fact, this fluctuation is influenced by hormonal changes, age, sexual practices, and antimicrobial drugs usage [14–17]. The microbial dysbiosis in vagina leads to overgrowth of opportunistic pathogens and ultimately contributes to the onset of disease [18].

Vaginal dysbiosis reflects the disruption of microbial community in vagina and is frequently associated with several gynaecological diseases. Multiple studies have shown the association between vaginal dysbiosis and increased vaginal infections such as bacterial vaginosis (BV), vulvovaginal candidiasis (VVC), sexually transmitted infections (STIs), i.e. trichomoniasis, human papillomavirus (HPV) infection, *Chlamydia trachomatis* (CT) infection, human immunodeficiency virus (HIV) susceptibility, and genital herpes infection [19–23]. One of the most prominent features of vaginal dysbiosis is the changes in vaginal pH. In a recent study, a significantly higher vaginal pH caused by decreased in lactate concentration was reported among BV, CT, and VVC patients as compared to healthy women [24]. The shift of microbial communities in vagina can also lead to severe gynaecological issues such as pregnancy loss, preterm labour, and low conception rates if left unattended [25]. Collectively, maintaining a harmonious balance of vaginal microbiota is crucial for a robust host-microbial interaction that promotes healthy vaginal ecosystem.

The knowledge advancement in human microbiota has accelerated the pace of new ventures in live biotherapeutics using beneficial microorganisms [26]. Previously, live biotherapeutics via faecal microbiota transplantation (FMT) has been proven successful in treating recurrent *Clostridioides difficile* infection [27]. Owing to the success of FMT, a similar approach using vaginal microbiota transplantation (VMT) could be effective in treating problematic vaginal infections. Recently, the first VMT has been reported to be able to reconstitute *Lactobacillus*-dominated microbiota with no observable adverse effects in recurrent-BV patients [28]. In addition, patients receiving *Lactobacillus* co-administered with antibiotics also showed reduced proneness towards recurrent BV [29]. In a similar study, a combined therapy using metronidazole with both *L. rhamnosus* GR-1 and *L. reuteri* RC-14 has successfully treated 88% of BV patients, as compared to 40% recovery rate for patients receiving only metronidazole treatment [30]. It has been suggested that these beneficial effects are partly associated with the cell surface-active molecules (SAMs) such as peptidoglycan (PG), lipoteichoic acid (LTA), biosurfactants (BS) and exopolysaccharides (EPS) [31, 32]. In fact, *Lactobacillus* SAMs has been proved to antagonise a plethora of bacterial and fungal pathogens such as *Candida albicans, Staphylococcus aureus, Streptococcus mutans*, *Escherichia coli*, *Pseudomonas aeruginosa*, and *Salmonella typhimurium* [33–35]. Therefore, further understanding in *Lactobacillus* and its derivatives (i.e. SAMs) could pave way for the development of novel remedy for infections caused by vaginal dysbiosis.

Over the past decade, investigations on vaginal microbiota have increased exponentially. These studies revealed the diversity of microbial communities that shaped up the distinct composition of vagina microflora in women [10, 24, 36–38]. The common findings from these studies suggested that *Lactobacillus*-dominated community is likely to be observed in the healthy-state vagina and higher vaginal pH (less acidic) is reported in diseased-state vagina. Besides, the microbial composition of vagina in some women are highly dynamic due to several predisposing host factors that eventually affects the host-microbial interaction. To date, the single root cause for vaginal dysbiosis should there be one remains to be identified. In this review, we seek to provide an overview of indigenous vaginal microbiota and mycobiota in women. Besides, we endeavour to underline the potential role of *Lactobacillus* and its derivatives (i.e. SAMs) in keeping vaginal pathogens under control to promote vaginal health.

## Indigenous vaginal microbiota in women

Healthy human vagina that is dominated by lactobacilli has been reported with marginal presence of fungi taxa [39]. Generally, beneficial bacteria communities coexist with human host in mutualism by protecting host vaginal milieu from colonisation of pathogenic microorganisms while the host provides nutrients for bacterial growth [11]. Colonisation and dominance of lactobacilli are essential traits of a healthy vaginal microbiota, commonly by species such as *Lactobacillus crispatus*, *Lactobacillus gasseri, Lactobacillus iners*, and *Lactobacillus jensenii* [10, 40, 41]. The changes in the microbiota composition of human vagina can occur through different life stages, this includes infant, puberty, pregnancy, and menopause stages [42]. In fact, hormonal changes, uncontrolled usage of antibiotics, menstruation, and vaginal douching are the common factors that steered the temporal changes in human vaginal microbiota [6, 43, 44].

**Fig. 1:**
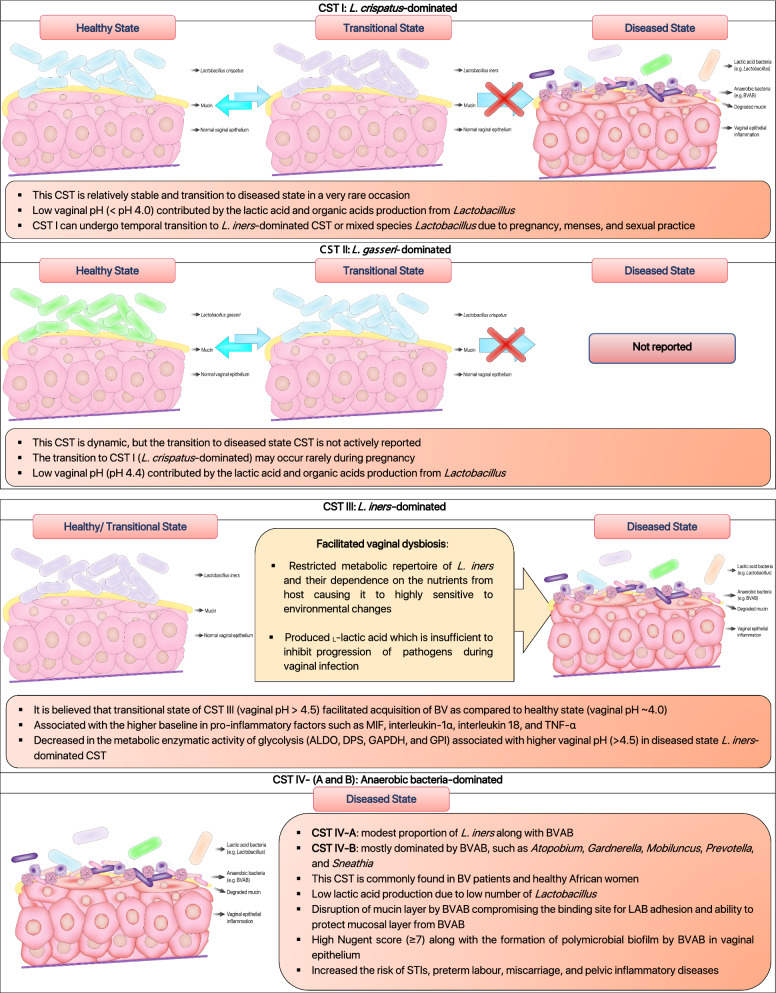

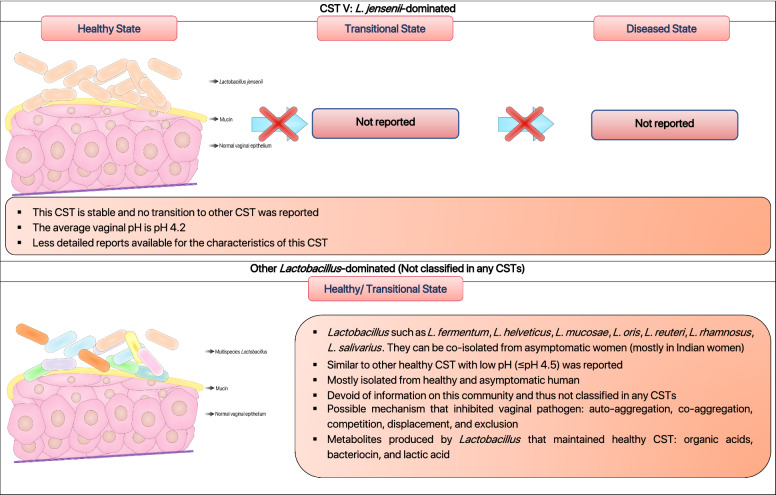
Schematic illustration of the human vaginal community state types (CSTs) based on scientific literature. The healthy and diseased state of vaginal microbiota can be classified into five common CSTs according to their respective characteristics. These CSTs are dominated mainly *L. crispatus*, *L. gasseri*, *L. iners*, bacterial vaginosis-associated bacteria (BVAB), and *L. jensenii* [6, 10, 47, 96, 262, 263]

Relative abundances of the predominant lactic acid bacteria (LAB) in healthy vagina determine the type of bacteria community groups, known as community state types (CSTs) [10]. The CSTs are grouped as CST I, II, III, IV, V, respectively with each of the CSTs is dominated by *L. crispatus*, *L. gasseri*, *L. iners*, polymicrobial flora including *Lactobacillus* and bacterial vaginosis-associated bacteria (BVAB), and *L. jensenii* (Fig. 1) [6, 10]. While CST I, III, and IV have been extensively studied and are commonly found in women, CST II and V, however, are rarely found in women [45, 46]. In fact, DiGiulo et al. [47] and van de Wijgert et al. [46], in their studies reported that vaginal microbiota from healthy women partly belongs to CST II and V. Gajer et al. [6] further characterised CST IV (lacks of significant abundance of particular *Lactobacillus* species) into subgroups CST IV-A and CST IV-B [6]. According to Gajer et al., CST IV-A generally contains a modest proportion of *L. iners* along with anaerobic bacteria such as *Corynebacterium, Finegoldia, Streptococcus, or Anaerococcus* whereas CST IV-B has a significant higher number of BVAB [6].

The presence of lactobacilli in vagina orchestrates a distinct inflammatory paradigm that contributed to distinct CSTs. It is noteworthy to mention that the presence of *L. iners* in CST III and CST IV were associated with higher baseline in pro-inflammatory factors such as macrophage migration inhibitory factor (MIF), interleukin-1α, interleukin 18, and tumour necrosis factor-alpha (TNF-α) which are responsible for the activation of inflammatory responses in vagina [48]. *Lactobacillus crispatus-*dominated vaginal microbiota (CST I) is always associated with healthy vagina, while *L. iners*-dominated vaginal milieu (CST III) is more prone to vaginal dysbiosis (Fig. 1) [49, 50]. Multiple studies have shown that the protective effect of *L. crispatus* against STIs, BV, and VVC, are intrinsically associated with the ability to produce lactic acid and bacteriocin that maintain the healthy state of vagina [51, 52]. Meanwhile, the lack of essential amino acids synthesis repertoire in *L. iners* has forced it to heavily rely on the exogenous amino acids derived from host [53]. Its restricted metabolic repertoire and dependence on the nutrients from host render it to be highly sensitive to environmental change [53]. Besides, it also produced a distinct isomeric form of lactic acid (L-lactic acid) which is insufficient to inhibit the progression of pathogens during vaginal infection [54, 55]. Additionally, a profusion of research have shown that human vaginal composition differs considerably between individuals, and greatly influenced by hormones (e.g. pregnancy and menses), as well as ethnicity [10, 56]. The influence of hormones particularly oestradiol, as a matter of fact, can stimulate the transition of CST I (*L. crispatus*-dominated) to CST III (*L. iners*-dominated) or mixed lactobacilli community, but rarely to diseased-state vaginal community (Fig. 1) [6, 46]. In addition, diseased-state (CST IV) and facilitated-BV state (CST III) vaginal community were more commonly found in sub-Saharan African [6, 10]. It is conceivable that genetic factors in these groups may alter the vaginal immune responses which favours the colonisation of *L. iners* and pathobionts that cause vaginal dysbiosis [56, 57]. As has been noted, the characterisation of microbial community in vagina has vastly extended our knowledge on the relationship between healthy and abnormal vaginal microbiota. The identification of prophage in *L. iners* genome indicated that bacteriophage could influence the adaptation strategies and abundance of lactobacilli in the vaginal ecosystem [58]. Thus, future studies are needed to elucidate the presence of *Lactobacillus* phage and its contribution to the healthy- and diseased-state vagina.

The core vaginal microbiota of majority Asian and white women is dominated by 80.2% and 89.7% of lactobacilli, respectively [10]. In contrast, *Lactobacillus* is not the sole genus that dominates vaginal microbiota in black and Hispanic women (only 59.6% and 61.9%, respectively) [10]. A cross-sectional study of 151 women (65 HPV-positive, 86 HPV-negative) revealed that HPV is significantly associated with higher abundance of anaerobes like *Bacteroides plebeius*, *Acinetobacter lwoffii*, and *Prevotella buccae* [59]. This finding implied that higher diversity of vaginal microbiota significantly increased the risk of HPV acquisition [59]. It is conceivable that disrupted vaginal microbiota may affect the host innate immunity against HPV infection that leads to development of cervical cancer [60]. In addition, Lee et al. [61] also revealed that vaginal dysbiosis is strongly interconnected with HPV acquisition. The vaginal microbiota of HPV-infected women has higher abundance of *Prevotella*, *Sneathia*, *Dialister*, and *Bacillus* with lower abundance of *Lactobacillus* as compared to healthy women [61]. Furthermore, disrupted vagina microbiota, characterised by low abundance of *Lactobacillus* and predominance of *G. vaginalis* was significantly associated with HPV acquisition and cervical neoplasia development [62]. Besides, low abundance of *Lactobacillus* and high proportions of *Gardnerella*, *Brucella*, *Sneathia*, and other miscellaneous bacteria in vagina were common among HPV- and genital warts-infected patients [63]. Taken together, the vaginal microbiota imbalance is strongly correlated with the risk of HPV-related infection. In short, the interventional treatment for vaginal dysbiosis could reduce the HPV acquisition and cervical cancer development [64].

In contrast to vaginal microbiota profiling, human vaginal mycobiota is still underexamined. The first high-throughput sequencing on vaginal mycobiota was only carried out in 2013 by Drell and her colleagues [39]. According to Drell et al. [39], 196 fungal operational taxonomic units (OTUs) were obtained from healthy Estonian women; the most dominant phyla was *Ascomycota* (58.0%), followed by unspecified fungal OTUs (39.0%), and *Basidiomycota* (3.0%). The most common OTUs that dominated phylum *Ascomycota* (order *Saccharomycetales)* are genus *Candida* (37.0%), mainly *C. albicans* (34.1%), *Candida krusei* (2.3%), *Candida alimentaria* (reported as *Candida* sp. VI04616 in this study) (0.3%), *Candida parapsilosis* (0.3%), and *Candida dubliniensis* (0.04%) [39]. Similarly, few studies also showed that *Candida* community are found in asymptomatic and healthy women [65, 66]. In addition, Ward et al. [67] reported that infants have an identical dominant mycobiota fungal species as the mother’s vagina (*C. albicans*) regardless of the methods of delivery [67]. Furthermore, *C. albicans* colonisation in infants is evident following vertical transmission from their mothers [68]. All in all, these findings indicate that *C. albicans* can colonise vagina without causing any symptomatic infections. At the same time, an increasing number of studies also highlighted risk factors such as hormones, diabetes, oral sex, intravaginal douching, self-treatment with antifungals and antibiotics, usage of intrauterine devices, and perineal laceration to be significantly associated with VVC occurrence [69–71]. Selected publications on human microbiome profiling on vaginal-related infections are summarised as in Table 1.

**Table 1:** Human vaginal microbiome study across the world (2007–2020) and its main findings

Country/place	Research design	Main findings	References
Tienen, Belgium	26 women: 11 healthy, 5 BV, 7 VVC, and 3 BV-VVC Age: 23–40 Cross-sectional study Microbial profiling by using PCR-denaturing gradient gel electrophoresis (PCR-DGGE) and real-time PCR analysis for 16S rRNA	PCR-DGGE revealed vaginal microbiota is stable over time in healthy women which dominated by *L. acidophilus*, *L. gasseri*, *L. iners*, *L. vaginalis* Low number of *G. vaginalis* co-exist with *Lactobacillus* in some healthy women which may acts as sentinel species and are susceptible to environmental, biological, and physical changes Low abundance of lactobacilli was reported in BV patients, concomitantly with an increase of BVAB such *G. vaginalis*, *A. vaginae*, *Leptotrichia, Megasphaera, Prevotella*, *Staphylococcus, Streptococcus, Veillonella* An increased of non-H_2_O_2_-producing *L. iners* and decreased of *L. acidophilus*, *L. gasseri*, *L. vaginalis* abundance in VVC patients	[264]
Iowa, USA	Cross-sectional study 42 women: 21 healthy and 21 RVVC-infected women (≥ 4 times in 2 years) Age: 18–40 Microbial profiling by using 16S rRNA terminal restriction fragment polymorphisms (T-RFLP)	No significant difference in bacteria communities and vaginal pH were reported among VVC-infected and healthy women Most RVVC patients were not symptomatic No correlation between vaginal communities and risk of RVVC was reported	[265]
Georgia and Maryland, USA	Cross-sectional study 396 non-pregnant women Age: 12–45 Microbial profiling by using barcoded 16S rRNA sequencing	Introduction of five vaginal CSTs (I, II, III, IV, V) to profile vaginal microbiota status based on lactobacilli abundance Higher vaginal pH (pH 4.7–5.5) has been reported for Black and Hispanic women in respect to Asian and White women (pH 4.2–4.4) Vaginal CST III (*L. iners*-dominated) and CST IV (BVAB-dominated) were more frequently reported in Black and Hispanic women	[10]
China	95 non-pregnant women: 30 healthy, 39 VVC, 16 BV-VVC, 10 BV Cross-sectional study Microbial profiling by using barcoded 16S rRNA sequencing	*Lactobacillus*-dominated vaginal microbiota is reported in healthy China women, with acidic vaginal pH (< pH 4.5) BV-infected women showed the highest diversity of vaginal microbiota (low abundance of *Lactobacillus*) BV-VVC women with a unique pattern of higher abundance of *Lactobacillus* Wide variety of vaginal microbiota reported in VVC-only women and showed unusual microbiota profile such as *Streptococcus*-dominated and *Gardnerella*-dominated microbiota after azole treatment BV-VVC women showed an increased abundance of *Lactobacillus* after antimicrobial treatment	[266]
Estonia	494 healthy and asymptomatic Caucasian women Age: 15–44 Cross-sectional study Bacterial profiling by barcoded 16S rRNA and fungal profiling by ITS sequencing	*Lactobacillus*-dominated vaginal microbiota reported in healthy and asymptomatic women BVAB such as *A. vaginae* and *G. vaginalis* is also reported in some women which can be classified as asymptomatic BV-infected women The diversity of vaginal microbiota increased with higher vaginal pH and malodorous discharge were present in women *Candida* species especially *Candida albicans* remains the most common yeast isolated from asymptomatic women	[39]
Seattle, USA	45 women enrolled in longitudinal study (2007–2010) Treatment with metronidazole for 7, 14, 21, 28 days Microbial profiling by using qPCR of 16S rRNA and bacterial dynamic analysis by mathematical modelling	Rapid reduction of BVAB within first day of treatment and gradual increment in *L. iners* abundance during the transient vacuum period The treatment is not effective against *G. vaginalis* and recurrence of BV is frequently reported in the patients	[267]
Toronto, Canada	182 pregnant women (11–16 weeks of gestation) Microbial profiles were compared with previous study non-pregnant Canada women (n = 310) Microbial profiling by using universal primer *cpn60* sequencing	Pregnant women with *Lactobacillus*-dominated CST has relatively higher abundance of lactobacilli as compared to non-pregnant women Lower richness and diversity (low abundance of Mollicutes and *Ureaplasma*) are reported in pregnant woman associated with low risk of preterm birth and pregnancy loss Hormone-induced glycogen production may provide conducive environment for bacterial growth in vagina explained pregnant women carried higher bacterial load as compared to non-pregnant women	[268]
Kenya, South Africa, Rwanda (Three sites)	80 women from Vaginal Biomarkers Study: 40 healthy and 40 BV 8 weeks longitudinal study (five consecutive visits) Gram-staining, qPCR, quantification of soluble immune mediators in cervicovaginal lavages	Total of 79% of the women with *Lactobacillus crispatus*-dominated microbiota are accompanied by *Lactobacillus vaginalis* whereas *L. jensenii* and *L. gasseri* are not present in the women Healthy women (normal Nugent score) has *L. iners*-dominated microbiota and is significantly associated with microbiota diversity and vaginal inflammation due to sexual activity and amenorrhoea BV-infected women (Nugent 7–10) reported low lactobacilli and high *G. vaginalis*, *A. vaginae*, and *P. bivia* abundance accompanied by the increased of proinflammatory cytokines (IL-1β, IL-12) and decreased of antiprotease elafin (IP-10)	[269]
University of Maryland, USA	40 non-pregnant women Cross-sectional study Microbial profiling by 16S rRNA sequencing Vaginolysin (cytototoxic protein) quantification	Higher concentration of vaginolysin was reported in CST-IV as compared to high abundance of CST-I microbiota Intermediate concentration of vaginolysin has been reported in *L. iners*-dominated microbiota (CST-III) women Higher abundance of *G. vaginalis* has been reported in lactobacilli-deficient vaginal microbiota and associated with increasing vaginal pH, Nugent score, and vaginolysin concentration	[270]
Istanbul, Turkey	28 healthy Caucasian women: 14 histologic-endometriosis and 14 healthy Prospective observational cohort study Microbial profiling by using 16S rRNA metagenomic sequencing	Lactobacilli remain the dominant genus in healthy and endometriosis-diagnosed women The abundance of *G. vaginalis* is significantly higher in endometriosis-diagnosed as compared to healthy women The absence of *A. vaginae* in vagina and cervix, increased of *E. coli*, *Shigella*, *Streptococcus*, and *Ureaplasma* abundance in cervix were reported in women with endometriosis	[271]
Centre for Health Behaviour Research, University of Maryland School of Public Health, USA	39 women: 26 HPV-positive (14 high-risk HPV) and 13 HPV-negative Cross-sectional study Microbial profiling by using 16S rRNA sequencing and vaginal metabolites profiling by using liquid chromatograph mass spectrometry	HPV-positive women were reported of having a higher biogenic amines (i.e. putrescine and ethanolamine) concentration and lower glutathione (GSH), glycogen, and phospholipid concentration compared to HPV-negative women Higher concentration of biogenic amines and glycogen-related metabolites were also reported in HPV-positive women (CST-III vaginal microbiota) Higher concentration of GSH, glycogen, and phospholipid-related metabolites have been reported in HPV-positive women (CST-IV vaginal microbiota) HPV-positive women had lower concentration of amines, lipids, and peptides as compared to HPV-negative women across all vaginal microbiota state Oxidative stress environments in vagina created from high level of biogenic amines and GSH may compromise host response against infection	[272]
Bologna, Italy	79 women: 21 healthy, 20 BV-, 20 CT-, 18 VVC-infected women Cross-sectional study Microbial profiling by using 16S rRNA MiSeq sequencing and metabolomic analysis by ^1^H-NMR	Vaginal microbiota in healthy women are dominated by *L. crispatus* Low abundance of *Lactobacillus* and high abundance of *A. vaginae*, *Faecalibacterium*, *Megasphaera*, *Roseburia* observed in CT-infected women Low abundance of *Lactobacillus* and high abundance of BVAB were reported in BV- and VVC-infected women Reduction of dimethylamine and increment of trimethylamine level in vaginal dysbiosis conditions (BV, VVC, CT) Production of lactic acid and branched-chain amino acids (i.e. valine, leucin, isoleucine) are higher in healthy women Increment of biogenic amines and short-chain organic acids were reported in BV-infected women Higher glucose level was reported in VVC-infected women, may decrease the abundance of *L. crispatus* and promote the virulence of *Candida*	[24]
Missouri (St. Louis), USA	255 women: 42 *Candida*-colonised and 213 non-*Candida* colonised Inclusion of Black and White women with normal, intermediate, and BV-type vaginal microbiota Nested cross-sectional study Microbial profiling by using qPCR of 16S rRNA Inhibition assay of lactobacilli against *Candida in vitro* growth	A total of 20% (52/255), 39% (99/255), and 38% (98/255) women reported *L. crispatus*-, *L. iners*-, and non-*Lactobacillus* dominated vaginal microbiota, respectively *Lactobacillus iners*-dominated vaginal microbiota is more likely to have *Candida* colonisation as compared to *L. crispatus-*dominated vaginal microbiota Cell-free supernatant from *L. crispatus* having lower pH (higher level of protonated lactic acid) and are correspondingly more effective to inhibit *Candida* colonisation as compared to *L. iners*	[273]
Kigali, Rwanda	68 high-risk BV or TV patients: only 55 actively sought for treatment Subjects treated with 7 days of 500 mg oral metronidazole Microbial profiling by using 16S rRNA HiSeq sequencing and BactQuant 16S gene quantitative PCR	The cure rate of BV after metronidazole treatment only achieved 54.5% Modest reduction in the abundance of BV-anaerobes after treatment (16.4% of total patients have reduction of 50% BV-population) Overall abundance of lactobacilli increased with *L. iners* recorded the highest abundance after treatment (success and failure) The presence of high abundance of pathobionts and *G. vaginalis* in women associated with likelihood of treatment failure potentially due to biofilm formation	[274]

BV: bacterial vaginosis; CT: *Chlamydia trachomatis*; RVVC: recurrent vulvovaginal candidiasis; VVC: vulvovaginal candidiasis; TV: *Trichomonas vaginalis*; BV-VVC: co-infection of BV and VVC; BVAB: BV-associated bacteria; CSTs: community state types; IP-10: Interferon-γ induced protein-10 (chemokine); ITS: Internal transcribed spacer; OTUs: Operational taxonomic units; PTB: Preterm birth; T1D: Type-I diabetes

*Candida. albicans* is the leading vagina coloniser and frequently isolated from vulvovaginal candidiasis (VVC) infected women [72, 73]. Vulvovaginal candidiasis happens in 75% of women at least once in a lifetime [72], while approximately 5–10% of women with the primary episode of VVC will develop RVVC (> four episodes annually) [74]. As one of the most common vaginal inhabitants, *C. albicans* has been frequently shown to co-colonise vagina with *Lactobacillus* [75]. Moreover, the isolation of non *C. albicans Candida* (NCAC) species such as *Candida tropicalis*, *C. glabrata*, *C. krusei*, *C. dubliniensis*, and *C. parapsilosis* were frequently observed in RVVC-infected women [76–79]. Non-specific symptoms reported by patients with VVC and recurrent VVC include vulvar erythema, pruritus, dyspareunia, burning sensations, white clumpy discharge, and soreness [72, 80]. Although VVC is not life-threatening, unresolved VVC affects their quality of life i.e. mental health, social life, sexual relationship, and working life [74, 81].

*Candida albicans* is a polymorphic yeast that is capable of yeast-to-hyphae morphogenesis under favourable conditions [82, 83]. Some plausible explanations on how *C. albicans* switches from mere coloniser to pathogen include vaginal dysbiosis, expression of virulence factors (i.e. hyphal and biofilm formation), and production of proteolytic enzymes [i.e. secreted aspartyl proteinases (SAPs)] that resulted in vaginal immune-toxicity [84]. Swidsinski et al. have demonstrated that intraepithelial lesions in VVC patients contained *C. albicans* hyphae accompanied by co-invasion of *G. vaginalis* and *L*. *iners* [85]. This is one the most compelling evidences showing that morphological plasticity that enables yeast-to-hyphae formation in *C. albicans* and the presence of BVAB could cause symptomatic VVC. Furthermore, disruption of vaginal microbiota (e.g. reduction of LAB population) may promote the ability of *Candida* species to invade vaginal epithelial cells [18]. Following the breach of vaginal epithelial cells, pseudohyphae and hyphae of *C. albicans* induced the NLRP3 inflammasome receptors of the epithelial cells through a cascade activation and ultimately triggered severe vaginal inflammation [86]. Of all the vaginal microbiota and mycobiota studies, *C. albicans* remains the most described causative agent for VVC [87]. The distinct hallmark of VVC are vaginal dysbiosis and vaginal mucosa inflammation caused by *Candida* species [85]. On top of that, the changes in vaginal mycobiota is proven to be associated with diabetes, pregnancy, immunodeficiency-allergic rhinitis, and recurrent vulvovaginal candidiasis (RVVC) status [88, 89]. As has been discussed, the microbiota and mycobiota interactions could contribute to VVC development in women through transient or continuous interplay between among them. Exploring these interactions and searching of potential microbial intervention are crucial to potentially prevent and treat VVC in women.

Bacterial vaginosis (BV) is the most common vaginitis among women of childbearing age and is characterised by significant changes in vaginal microbiota composition from a *Lactobacillus*-dominated to a polymicrobial community [24, 90]. According to Peebles et al., 23 to 29% of women population across seven regions were infected with BV and this has caused a massive economic burden of USD 3.7 to 6.1 billion per annum globally [91]. Bacterial vaginosis can be diagnosed by Amsel criteria, Gram staining, Nugent score, and molecular assays [40, 92]. It is usually accompanied by a significant number of *G. vaginalis*, *Prevotella* species, *A. vaginae*, *Sneathia* species, and other BVAB as a result of disrupted vaginal microbiota [93, 94]. Frequently, BV is associated with elevated risk of HIV acquisition, miscarriage, pelvic inflammatory diseases, preterm labour, postpartum endometritis, and STIs acquisition [90, 95–97]. Besides, BV eventually causes significant psychosocial stress on women. Bilardi et al. [98] demonstrated that women with recurrent BV often experience embarrassment, low self-esteem, and frustration in their daily life.

It is conceivable that the production of bacteriocin and lactic acid from *Lactobacillus* inhibit the over-proliferation of BVAB in the vagina [99]. However, *Lactobacillus*-dominated vaginal microbiota is displaced by the overgrowth of *Gardnerella* species and other BVAB when vaginal dysbiosis happens [100]. Recent studies have demonstrated that the synergistic interactions between BVAB such as *G. vaginalis* and *A. vaginae* significantly enhanced the severity of BV by increasing bacterial burden [101, 102]. Another important feature of BV is polymicrobial biofilm formation mainly by *G. vaginalis*, while the presence of other co-colonised BVAB was shown to enhance the biofilm thickness of *G. vaginalis* growth [85, 103–105]. Several studies have also demonstrated that BV-associated vaginal microbiota with reduced number of *Lactobacillus* increased the incidences of other STIs [106–108]. Cone [109] inferred that *Lactobacillus*-dominated microbiota reduces the transmission of STIs by strongly acidifying the vagina milieu and lowering inflammatory cytokines. Multiple studies also consistently showed that the presence of *Lactobacillus* significantly reduced *C. trachomatis* virulence via lactic acid [54, 110, 111].

Sexually transmitted infections (STIs) such as chlamydia infections (mainly caused by *C. trachomatis*), gonorrhoea (*Neisseria gonorrhoeae*), trichomoniasis (*Trichomonas vaginalis*) and syphilis (*Treponema pallidum*) often engendered severe forms of cervicitis, urethritis, vaginitis and genital ulceration in women [112–114]. According to World Health Organization (WHO), the annual global estimate for STIs was 376.4 million (chlamydia infections: 127.2 million; gonorrhoea: 86.9 million; syphilis: 6.3 million; trichomoniasis: 156.0 million) [112]. Generally, STIs are curable with short regimens of antibiotic treatment. However, STIs can be transmitted to others and cause epidemic if left untreated [115]. These STIs are commonly correlated with high risk of cervical cancer, infertility, preterm labour, and pelvic inflammatory disease in women [114, 116, 117]. Numerous studies have consistently shown that disrupted or BV-associated vaginal microbiota (low-*Lactobacillus* abundance) increased STIs incidences [106–108, 118–120]. Besides, the occurrence of STIs is associated with high risk of human immunodeficiency virus (HIV) acquisition. Galvin and Cohen [121] have shown that STIs are able to disrupt the mucosal layer and immune homeostasis of vagina, resulted in increased of HIV shedding [121]. At the same time, asymptomatic *Chlamydia trachomatis* infection is often under-diagnosed and left untreated among infected individuals [122]. A balanced vaginal microbiota that is rich with *Lactobacillus* is able to modulate vaginal epithelial cell proliferation and d- lactic acid production and subsequently reduced *C. trachomatis* elemental bodies internalization into epithelial cells [54]. Therefore, these studies highlighted the importance of vagina homeostasis in providing a natural barrier against the vaginal infections.

Vagina also serves as a reservoir for urinary tract infections (UTIs)-causing uropathogens in women [123]. The commonest pathogens that cause UTIs are *Escherichia coli*, *Klebsiella pneumoniae*, *Staphylococcus epidermidis*, *Streptococcus agalactiae* (group B *Streptococcus*), *Enterococcus faecalis*, *Proteus mirabilis*, and *Pseudomonas aeruginosa* [124–126]. While UTIs are curable by antibiotics, severe complications including pyelonephritis, haematuria, and chronic kidney disease (CKD) can cause permanent kidney damage [127, 128]. Studies have shown that pathogens such as *Gardnerella*, *Prevotella*, and *Ureaplasma* potentially ascended from vaginal tract before causing infection in the urinary tract via the urethra and urinary bladder [129–131]. Vaginal dysbiosis has been shown to increase the risk of UTIs acquisition as compared to *Lactobacillus*-dominated vaginal microbiota [123, 132]. In fact, exposure of mice vagina to *G. vaginalis* triggered the recurrent UTIs that are caused by *E. coli* [133]. Thus, maintaining vaginal homeostasis could suppress the pathogenesis of uropathogens in the urinary bladder. Another serious illness that torment women of child-bearing age is toxic shock syndrome (TSS), which is associated with the colonisation of TSS toxin (TSST-1) producing *S. aureus* in vagina [134]. It is well established that TSST-1 is produced in neutral pH (i.e. pH 6.5–7.0), a condition which is frequently reported in diseased-state vagina [135]. Multiple studies have shown that usage of menstrual cups, tampons, and contraceptive diaphragms can disrupt *Lactobacillus*-dominated vaginal microbiota and enhance the growth of *S. aureus* and production of TSST-1 [136–138]. The over-production of TSST-1 can lead to severe complications such as organ failure, systemic inflammation, and death in women [139]. In summary, vaginal dysbiosis can cause the loss of LAB protective effect in vagina and increase the risk of uropathogens ascending from vagina that eventually lead to UTIs.

A well balanced and disrupted vaginal microbiota essentially are significantly associated with healthy- and diseased-state vagina. Apart from host predisposition to BV, STIs, VVC, and UTIs, disruption of vaginal microbiota actively deprives the beneficial functions of *Lactobacillus* against opportunistic pathogens in vagina. Further exploration with a holistic study design such as diverse populations, ethnicity, and geographical area can potentially lead to development of predictive marker for diagnosis of disrupted vaginal microbiota. The complementary approach in improving and restoring vaginal microbiota to non-diseased status is thereafter needed by using biotherapeutic agents such as *Lactobacillus* to reduce risks of these vaginal infections.

## Potential of *Lactobacillus* in keeping vaginal pathogens commensals

Lactic acid bacteria are representative microorganisms from a diverse group of bacteria that are characterised as Gram-positive, microaerophilic, acid-tolerant, non-sporulating, and capable of lactic acid production [140, 141]. The prevailing genera of LAB that are used as probiotics are *Lactobacillus*, *Bifidobacterium*, *Streptococcus*, *Enterococcus*, and *Pediococcus* [142, 143]. The GRAS status of lactic acid by Food and Drug Administration (FDA) has been extensively utilised in food, dairy, and pharmaceutical industries [144, 145]. For instance, *Lactobacillus delbrueckii* subsp. *bulgaricus* has been employed along with *Streptococcus thermophilus* as starter culture in manufacturing yoghurts and cheeses [146, 147]. According to Reid et al., the administration of probiotic lactobacilli in adequate amounts is able to confer health benefits to host by restoring microbial and host immune homeostasis [148].

An increasing number of studies have elucidated the fundamental probiotic effects of *Lactobacillus* against pathogens present in the GI tract, oral cavity, vagina, and epidermal layer [149–152]. *Lactobacillus acidophilus* KS400 has been proven to produce bacteriocin through fermentation and inhibited the growth of urogenital pathogens such as *G. vaginalis*, *S. agalactiae*, and *P. aeruginosa* [153]. Additionally, bacteriocin from vaginal *L. rhamnosus* (Lactocin 160) was able to produce transient pores on the cytoplasmic membrane of *G. vaginalis* by collapsing the chemiosmotic potential of the pathogen [154]. Multiple studies have also shown that aerobic vaginitis (AV)-causing pathogens such as *E. coli*, *E. faecalis*, *S. aureus*, *S. epidermidis*, and *S. agalactiae* commonly reside in the vagina and induce inflammatory vaginitis [155, 156]. Prolonged treatment of vaginitis with antimicrobial drugs can result in development of drug resistance [157, 158]. Thus, the probiotic lactobacilli-based approach as an alternative to the conventional antimicrobial treatment is being extensively studied. According to Bertuccini et al. [159], *L. rhamnosus* HN001 and *L. acidophilus* GLA-14 were able to significantly inhibit the growth of *G. vaginalis*, *A. vaginae*, *S. aureus*, and *E. coli*. In an attempt to elucidate the effect of *Lactobacillus* introduction in vaginal microbiota, a study has shown that oral administration of mixed *L. acidophilus* La-14 and *L. rhamnosus* HN001 have significantly increased vaginal *L. rhamnosus* and *L. acidophilus* abundance starting at day 7 and 14, respectively [160]. In a similar study, orally-administered of probiotic formulations (*L. acidophilus* PBS066 and *L. reuteri* PBS072) and (*L. plantarum* PBS067, *L. rhamnosus* PBS070 and *B. lactis* PBS075) significantly increased the abundance of lactobacilli and bifidobacteria in vagina, starting at day 7 as compared to placebo-control group [161]. Apart from that, it has been reported that culture supernatants from multiple lactobacilli strains inhibited *C. albicans* significantly by suppressing the expression of adhesion and hyphae-related genes [162]. Ironically, genes related to SAPs were not affected, thus suggests the importance of these proteinases in the survival of *C. albicans* within *Lactobacillus*-dominated vagina. The anti-*Candida* activity observed was partially attributed to the presence of bacteriocin, hydrogen peroxide, and lactic acid [162]. In addition, Li et al. implicated that both *L. crispatus* and *L. delbrueckii* were able to inhibit 60 to 70% of *C. albicans* in VVC Sprague-Dawley rat model as compared to non-treated control [163].

*Lactobacillus* interventions have been demonstrated to be beneficial in co-treatment with antimicrobials drugs and prevention of recurrent infections. One of the studies that adopted this approach showed that oral co-administration of multispecies-lactobacilli (*L. fermentum* 57A, *L. gasseri* 57C, and *L. plantarum* 57B) with metronidazole, lengthened the relapse of BV (51%) and AV (71%) significantly, and maintained the acidity of vaginal pH [164]. It is believed that the bile acid-tolerant *Lactobacillus* were able to increase the abundances of lactobacilli in intestinal before migrating to vagina cavity [148, 165]. Nevertheless, the exact mechanism of how oral probiotics travel and dominate in vagina remains debatable [166, 167]. Intravaginal administration of probiotics was also invented to restore the disrupted vaginal microbiota. Bohbot et al. [168] reported that 28 days intravaginal administration of lyophilised *L. crispatus* IP 174178 was able to reduce the recurrence rate (20.5%) and prolonged the time for BV recurrence (28%) as compared to placebo-control group. Moreover, vaginal tablet consists of *L. fermentum* LF15 and *L. plantarum* LP01 restored the acidity of vaginal pH and the threshold level of Nugent score to below 7 (balanced vaginal microbiota) through the inhibition of *G. vaginalis* [169]. *Lactobacillus rhamnosus* BMX 54 also has been clinically tested on BV patients and has been shown to be able to restore vaginal microbiota to balanced state following three months administration [170]. In addition, *L. rhamnosus* BMX 54 also showed its potential to be used as adjuvant treatment in reshaping vaginal microbiota and by reducing BV recurrence after six to nine months of treatment [171]. Recent evidence showed that intermittent application of vaginal capsule (containing *Lactobacillus acidophilus* W70, *Lactobacillus brevis* W63, *Lactobacillus helveticus* W74, *Lactobacillus plantarum* W21, *Lactobacillus salivarius* W24, and *Bifidobacterium bifidum* W28) restored *Lactobacillus*-dominated vaginal microbiota and significantly reduced risk of BV incidence by 2.8-fold as compared to non-treated control group (10.18 per person-year) [172]. Meanwhile, the usage of lactobacilli could also reduce the rate of VVC recurrence. For instance, oral co-administration of clotrimazole and oral capsule (containing *L. acidophilus* GLA-14 and *L. rhamnosus* HN001) with bovine lactoferrin RCX were shown to significantly reduce VVC recurrence by 58.4% and 70.8%, respectively at three and six months as compared to non-lactobacilli administration control group [173]. It is imperative that the restoration of vaginal microbiota could prevent various vaginal infections and the rate of its recurrence. According to Xie et al. [174], there are insufficient shreds of evidence to recommend the usage of only probiotics in countering VVC and BV as compared to conventional drugs treatment.

A eubiotic vaginal ecosystem that is dominated by *Lactobacillus* has the potential to protect host against HIV and STIs [20, 175]. According to McClelland et al., high abundance of BVAB was associated with the risk of HIV acquisition in women [108], possibly due to increased vaginal pH and production of an enzyme that inhibits anti-HIV immunity [176]. Several studies have been carried out to determine the potential of *Lactobacillus* in suppressing BV-associated bacteria and HIV transmission in vitro and ex vivo [177, 178]. Culture supernatant produced by vaginal-isolated *Lactobacillus* strain has been shown to be able to suppress HIV-type 1 infection in human cervicovaginal tissue [178]. In this study, the *Lactobacillus* culture supernatant has been proven to be viricidal and it helps in reducing virion dissemination in the host [178]. Besides, heat-killed *L. gasseri* also demonstrated high inhibitory activity (81.5%) against HIV-1 strain X4 infectivity on TZM-bl cellosaurus cell line [179]. In a similar study, *L. casei* 393 (1 × 10^4^ cells/mL) was able to inhibit HIV-1 pseudoviruses (AD8, DH12, and LA1), ranged from 60 to 70% after 30 min of co-incubation [180]. Recently, Palomino et al. found that the inhibitory effect of *Lactobacillus* against HIV-1 infection is associated with the presence of extracellular vesicles, which inhibit the HIV adhesion and viral entry to target cells [181]. Prospective studies have consistently suggested that disrupted vaginal microbiota increased the risk of HIV acquisition among women [23]. Future studies should prioritise the elucidation of mechanisms that explain the vaginal dysbiosis and HIV acquisition, and the discovery of probiotic lactobacilli as effective intervention for HIV prevention.

Collectively, *Lactobacillus* shows a promising effect in prevention of vaginal infection such as BV and VVC. The complementary approach by using probiotic lactobacilli to redress vaginal microbiota imbalance is in dire need to reduce the utilisation of antimicrobial drugs. More clinical trials on the efficacy of probiotic lactobacilli against vaginal infection should be conducted to address the heterogeneity of probiotic effectiveness.

## Potential of surface-active molecules (SAMs) from *Lactobacillus*

Many potential mechanisms have been proposed to be responsible for the probiotic effects of lactobacilli, these include competition for colonisation, modulation of host immune response, cross-feeding of beneficial microbiota, production and secretion of lactase, bile salt hydrolase, organic acids and antimicrobial compounds [Reviewed in [182, 183]]. The probiotic characteristics of lactobacilli associated with the host-*Lactobacillus* interaction is reckoned to be contributed by the *Lactobacillus* surface-active molecules (SAMs) [184]. *Lactobacillus* SAMs that have been reported to support probiotic actions are peptidoglycan (PG), bacterial polysaccharides, biosurfactants (BS), and teichoic acids (TA) [185, 186]. The core SAMs that are shared among LAB includes lipoteichoic acid, polysaccharides, surface layer associated proteins (SLAPs), mucin-binding proteins (MUBs), and fibronectin-binding proteins [187]. This core SAMs govern the host-microorganism interactions upon LAB adhesion. In fact, it has been shown that SAMs mediate the host physiological responses directly via direct adherence to the epithelial cells and pattern recognition receptors (PRRs) on mucosa layer [187]. As *Lactobacillus* SAMs could be important for the regulation of host-microorganism interaction in vagina, research on these SAMs should be focused to a greater extent in order to produce novel SAMs-based treatment, potentially as an alternative to currently available therapeutic options.

## Peptidoglycan (PG)

Peptidoglycan (PG) is a biopolymer which comprises glycan strands connected by *N*-acetyl-glucosamine (GlcNAc) and *N*-acetylmuramic acid (MurNAc) side chains that form the cell surface of Gram-positive bacteria such as *Lactobacillus* and bifidobacteria [188, 189]. Generally, the cytoplasmic membrane of *Lactobacillus* is surrounded by PG network and other biopolymers, namely teichoic acids (TA), S-layer proteins, and polysaccharides [189, 190].

In general, STI pathogen such as *N. gonorrhoeae* is able to suppress the host Th-1-driven adaptive immune response by inhibiting the production of interleukin-12 (IL-12) [191]. In view of that, intravaginal administration of microencapsulated IL-12 was able to reverse the immunosuppression in mice and also promotes the clearance of gonorrhoea infection [191]. At the same time, *Lactobacillus* PG demonstrates outstanding immunomodulatory activity in improving host innate immune responses. For instance, *Lactobacillus casei* PG was able to induce the production of IL-12 by mice peritoneal macrophages through toll-like receptors 2 (TLR2) and nucleotide-binding oligomerization domain 2 (NOD2) [192]. Moreover, *L. plantarum* CAU1055 PG demonstrated the ability to ameliorate nitric oxide-induced inflammation in RAW264.7 murine macrophages through the inhibition of nitric oxide (NO) synthase, cyclooxygenase-2 (COX-2), and cytokines (TNF-α and interleukin-6) [193]. In a similar study, PG derived from *L. acidophilus* has been reported to significantly reduce the NO synthase and COX-2 levels on LPS-induced RAW 264.7 macrophages as well [194]. On top of that, vaginal isolate *L. crispatus* PG was able to stimulate the expression of CD207 of Langerhans cells (antigen-presenting dendritic cells on vagina) and significantly reduced the expression of receptors for HIV entry [195]. The balance of vaginal microbiota and immune system in vaginal epithelial cells are crucial to prevent vaginal infection [196]. The potential effect of PG in modulating immune homeostasis could effectively assist in the maintenance of healthy vaginal ecosystem for women’s health and well-being. Apart from the reported immunomodulatory activity, *L. brevis* PG also exhibited strong antiviral activity against genital herpes simplex virus-2 (HSV-2) [197]. According to Mastromarino et al. [197], the antiviral activity of *L. brevis* PG was unaffected by heat- and protease-treatment, and it still inhibited HSV-2 replication significantly in a concentration-dependent manner.

## Lipoteichoic acid (LTA)

*Lactobacillus* PG is usually decorated with teichoic acids (TA) or lipoteichoic acids (LTA) [198]. Lipoteichoic acids are generated from the polymerisation of glycerol-phosphate and are bound to the cytoplasmic membrane [199, 200]. Together with other SAMs, LTA modulates the host pattern recognition receptors (PPRs) and several signalling pathways of host that are accounted for the probiotic and anti-pathogen effect of *Lactobacillus* [185]. The eradication of polymicrobial biofilms in human vagina is one of the strategies that can be used to impede bacterial virulence and prevent the onset of BV [201]. *Lactobacillus plantarum* LTA hampered the formation of *S. mutans* biofilms on hydroxyapatite discs via the attenuation of the sucrose decomposition [35]. Moreover, *L. plantarum* LTA significantly inhibited *E. faecalis* biofilm formation and preformed biofilm on human dentin slices suggesting that LTA can be employed as a preventive and therapeutic measures for *E. faecalis* infection [202]. Also, *L. plantarum* LTA inhibited polymicrobial biofilm consists of *Actinomyces naeslundii*, *Lactobacillus salivarius*, *E. faecalis*, and *S. mutans* on dentin slices [203]. Other than anti-adhesion and anti-biofilm properties, *Lactobacillus* LTA also possesses immunomodulatory activity. For instance, *Lactobacillus johnsonii* La1 and *Lactobacillus acidophilus* La10 LTAs ameliorated the overstimulation of pro-inflammatory cytokines production such as TNF-α, IL-8, and interleukin-5 (ENA-78) in intestinal epithelial cells, in the presence of lipopolysaccharides (LPS) or Gram-negative bacteria [204]. According to Ahn et al., LTA from *L. plantarum* K8 also modulated inflammatory cytokines (TNF-α and IL-10) production in LPS-challenged THP-1 cells [205]. Patients with BV, sexually-transmitted diseases are often associated with overstimulation of pro-inflammatory cytokines and neutrophils recruitment to vagina mucosa surface [119, 206]. Thus, the immunomodulatory activity of lactobacilli LTA could dampen the overstimulation and vaginal inflammation caused by pathogens.

## Bacterial polysaccharides

Bacteria form tightly-linked polymers on the cell surface and release them to the environment as exopolysaccharides (EPS) (loosely unattached slime) [31, 207]. By exploiting the surface polysaccharides in mimicking host’s glycan structure, pathogenic bacteria are able to evade the host immune system during colonisation [208]. Generally, EPS secreted by bacteria are crucial for the adhesion and cellular recognition during host-microorganism interaction [209]. Exopolysaccharides are high molecular weight, biodegradable carbohydrate polymers and are designated into homopolysaccharides or heteropolysaccharides based on their monosaccharides constituents [Reviewed in [210–212]]. Exopolysaccharides from LAB have attained substantial attention in the past decade due to their capability to inhibit bacterial toxins produced by *Bacillus cereus* [213]. The production of *Lactobacillus* EPS is regulated by the culture conditions and nutrient compositions during fermentation [214, 215]. For instance, the production of *L. rhamnosus* EPS was significantly increased by 40 to 50% following 48 h of co-fermentation with *S. cerevisiae*, owing to the upregulation of EPS operons expression that enhanced amino acid biosynthesis, carbohydrate metabolism, and fatty acid metabolisms [216]. Besides, the production of *Lactobacillus pentosus* EPS was strongly affected by different carbon sources used for the fermentation process such as glucose which enhance the viscosity of EPS for a better thickening effect in milk production [217]. Similar study also reported that higher production of *L. plantarum* EPS was observed in de Man, Rogosa and Sharpe (MRS) medium supplemented with glucose as carbon source [218]. Different carbon source of culture medium also significantly influences the functional activity of *Lactobacillus* EPS. As proof, MRS supplemented with sucrose significantly increased the EPS production of *L. plantarum* LPC-1 [219]. However, higher antioxidant activity was reported in *L. plantarum* LPC-1 when glucose is used as the carbon sources as compared to sucrose [219]. Collectively, the two concomitant factors that affect the EPS production (*Lactobacillus* strains and carbon source) resulted in distinct rheological properties of EPS which can influence the EPS functional activity.

The unique physiochemical properties of *Lactobacillus* EPS has the potential to confer health benefits to human as it has been reported to possess anti-atherosclerotic, anticancer, antioxidant, antiviral, anti-yeast, immunomodulatory, and prebiotic properties [220–224]. Human defensin-2 is an antimicrobial peptide that is secreted by the host epithelial cells which helps in the regulation of inflammation and microbiota function in vagina tract [225]. Correspondingly, *Lactobacillus crispatus* L1 EPS strongly enhanced the ability of vaginal epithelial VK2 cell to produce human defensin-2 protein (candidacidal effect) and it also reduced the adhesion of *C. albicans* (48%) by competitive exclusion [226]. Likewise, the competitive exclusion was observed between *L. rhamnosus* GG EPS and multiple *Candida* species, as demonstrated by the significant reduction of the adhesion of *C. albicans* (30%) and *C. glabrata* (25%) on VK2 and human bronchial Calu-3 cell line, respectively [224]. It is perceivable that the yeast-to-hyphae switching is crucial for *C. albicans* pathogenesis and immunopathogenesis [227]. A study conducted by Allonsius et al. [224] reported that EPS from *L. rhamnosus* GG inhibited hyphal formation in *C. albicans* by 40% further corroborates the potential anti-*Candida* properties of *Lactobacillus* EPS. Bacterial vaginosis is characterised by the presence of polymicrobial biofilm on the vaginal epithelia [228]. *Lactobacillus plantarum* WLPL04 EPS was reported to significantly reduce the adhesion of *E. coli*, *P. aeruginosa*, *S. aureus*, and *Salmonella typhimurium* on HT-29 cell line [33], which make it a potential anti-biofilm agent that is worth to be developed for better BV management.

The occurrence of BV has been associated to the formation of high oxidative stress (e.g. high level of malondialdehyde (MDA) production and low superoxide dismutase (SOD) activity) and degradation of mucin in the vaginal milieu [229, 230]. Thus, it is crucial that high antioxidant capacity of vaginal epithelium may reduce the oxidative stress formed during the BV infection and enhance the vaginal immune system against pathogens. *Lactobacillus plantarum* C88 EPS was also shown to demonstrate high antioxidant effects by reducing MDA level and raising SOD activity in a dose dependent manner [231]. Besides, pure EPS extracted from *L. helveticus* KLDS1.8701 significantly improved the antioxidant activity of mice liver towards oxidative stress through the reduction of SOD activity [232]. Apart from antioxidant capability, *Lactobacillus* EPS also demonstrated the ability to ameliorate degraded mucin barrier in cell. For instance, *L. plantarum* EPS promotes mucosal healing and protection by increasing the mucin MUC2 gene expression, tight junction protein expression and goblet cell differentiation in mouse colon [233, 234]. In fact, mucin has been shown to prevent the adhesion of vaginal pathogens and promotes the adhesion of LAB on epithelial cells [25].

The prebiotic properties of EPS have been actively explored. Generally, prebiotics can be retrieved from natural sources to serve as an energy source for epithelial cells and stimulate the growth of beneficial bacteria in the gut [142]. The general requirements for a compound to be considered as “prebiotic” include the ability to withstand gastrointestinal enzymes and absorption in small intestines, as well as the capability to stimulate the metabolic activities of beneficial bacteria via fermentation [212]. Sims et al. [235] have shown that the utilisation of prebiotics oligosaccharides such as β-glucan, inulin, and fructo-oligosaccharides stimulated the growth of probiotic LAB, suggesting the combination of prebiotic and probiotic can offer health benefits to host. The glucan EPS of *Lactobacillus brevis* ED25 was demonstrated to increase the shelf life of probiotic *L. rhamnosus* GG in food and also elevated the viability of *L. rhamnosus* GG [236]. Polysaccharides from probiotic EPS were suggested to play a role in elevating the abundance of normal flora in intestinal surface through bacteria cross-talk [237]. Extrapolation can be made that similar interaction could be observed in the beneficial bacteria within the vaginal milieu.

## Biosurfactant (BS)

Biosurfactants (BS), also known as bio-emulsifier are amphipathic active compounds mostly synthesised by microorganisms [238]. These amphipathic molecules have granted microorganisms the ability to reduce the surface and interfacial tension between aqueous solution through emulsion [239]. Biological BS can be categorised into low molecular weight surfactant (e.g. glycolipids and lipopeptides) and high molecular weight surfactant (e.g. glycoprotein complexes, lipopolysaccharides, and lipoproteins) [240]. Besides its important role in agriculture, animal feeds, cosmetic, food and oil industries, BS has recently drawn the attention of scientific community due to its bioremediation potential [241, 242]. However, the functional activity of *Lactobacillus* BS remains understudied [240]. Mouafo et al. has shown that the production of BS from *Lactobacillus* is dependent on the choice of fermentative carbon sources. Carbon sources originated from sugarcane and glycerol increased the yield of BS effectively compared to MRS broth [243].

Biosurfactants have been reported to exhibit anti-adhesion and antimicrobial characteristics by altering the surface chemistry for microbial attachment [182]. Sophorolipid is a type of glycolipid BS commonly produced by non-pathogenic yeast *Starmerella bombicola* [244]. This compound has been proven to be able to inhibit the formation of *C. albicans* biofilm, as well as disrupt the preformed *C. albicans* biofilms [244]. Astonishingly, Haque et al. [244] also found that the combination of sophorolipid and antifungal drugs is highly effective in inhibiting *C. albicans* as shown by the absence of *C. albicans* hyphae and biofilm complex networks after treatment. As for BS produced by *Lactobacillus*, it was recently reported that BS from *L. acidophilus* ATCC 4356, *Lactobacillus debrueckii* ATCC 9645, and *Lactobacillus paracasei* 11 significantly reduced the biofilm formation of vaginal pathogen *C. albicans* by 40 to 50% [245]. Also, BS derived from *L. brevis* CV8LAC was reported to effectively inhibit 24 h-, 48 h-, and 72 h-*C. albicans* biofilm formation on silicone elastomeric discs (~ 90%) [246]. Other than inhibition on *C. albicans* biofilm, BS from *L. jensenii* P_6A_ and *L. gasseri* P_65_ also exhibited potent antimicrobial and anti-biofilm activities against multiple urogenital pathogens such as *E. coli*, *Klebsiella pneumoniae*, *Staphylococcus saprophyticus*, and *Enterobacter aerogenes* [247]. Besides, crude *L. paracasei* BS inhibited *Streptococcus pyogenes, Staphylococcus epidermidis*, *E. coli*, *P. aeruginosa*, *S. aureus*, and *S. agalactiae* [248]. Gudiña et al. [248] found that *L. paracasei* BS is still highly stable following pH alkalisation (pH 6 to 10) and heat treatment (60 °C). On top of that, the extraction of crude *L. paracasei* BS via acidic preparation also greatly enhanced the antimicrobial activity [248]. Multiple studies were also performed to identify the effects of lactobacilli BS against other vaginal and uropathogens. According to Spurbeck and Arvidson [249], *L. gasseri* 33323 BS demonstrated anti-adhesion activity against sexually-transmitted pathogen *Neisseria gonorrhoeae* through the blocking of fibronectin, an extracellular matrix component on the epithelial cells. Biosurfactant from *L. crispatus* also significantly inhibited *N. gonnorrhoeae* growth (> 50%) following incubation at two timepoints, i.e. 7 min and 60 min [250]. Jiang et al. have reported that the mechanism involved in the anti-adhesion activity of *L. helveticus* 27170 BS against *S. aureus* was associated with the disruption of autoinducer-2 signaling (quorum sensing molecule) in *S. aureus* [251]. Besides, Satpute et al. revealed that *L. acidophilus* BS was able to reduce *E. coli*, *S. aureus, P. vulgaris*, *B. subtilis*, and *P. putida* biofilms on medical implant polydimethyl siloxane (PDMS) surface through anti-adhesion mechanism [252]. Recently, BS from *L. crispatus* BC1 also demonstrated significant in vitro anti-adhesion activity against *C. albicans* through exclusion mechanism on human cervical cancer HeLa cell line and in vivo immunomodulatory activity by reducing the leukocyte influx (i.e. prevent mucosal damage) caused by *C. albicans* in mice [253]. Based on these findings, it is conceivable that the mechanism of action of *Lactobacillus* BS involves adhesion interruption rather than killing of the invading pathogens.

## Challenges of *Lactobacillus* SAMs applications

Although there are substantial evidences that *Lactobacillus* SAMs could benefit human, implementation their use still remain obscure and challenging. One such challenge would be the cost required for their production. As their extraction is often hindered by low yields, optimisation of growth medium composition and extraction methods are essential [210, 215, 254]. In addition, other factors such as type of carbon sources and pH also significantly influence EPS structure and yields [255]. Therefore, mass production of SAMs often requires extra efforts that could be time-consuming and cost-ineffective. Additionally, the adoption of SAMs in large-scale industrialisation remain elusive due to the nature of SAMs structure fluctuation according to the medium composition. In an attempt to lower the high production cost of fermentation to produce lactobacilli SAMs, carbon-rich agricultural wastes such as bran, sugar cane and beet molasses could be employed as an alternative culture medium [256]. The utilisation of low-cost agricultural waste-based media potentially reduce the high cost inputs during large-scale fermentation process and meet the high market demand of lactobacilli SAMs in the future. Moreover, the cost of lactobacilli SAMs production can be reduced by determining the most economical culture medium composition for the maximum yield [215]. For instance, economic modelling via formula adjustment and production possibility curve (PPC) were performed to assess the carbon source for optimal culture medium composition and optimal productivity set (OPS) of *L. acidophilus* EPS production [215]. According to Lin et al. [215], the total cost production of *L. acidophilus* EPS by using MRS-nutrient broth culture medium was able to reduce by 30% (USD 7.5/kg/L culture) as compared to MRS only (USD 11.0/kg/L culture). Apart from that, statistical design is also one of the salient approaches that can be utilised to optimise media composition for the maximum yield of lactobacilli SAMs. The most widely used statistical designs for the optimisation of media suitable for SAMs (e.g. biosurfactant) production are factorial design and Response Surface Methodologies (RSM) [257]. In fact, factorial design such as Plackett–Burman Design (PBD) has been employed to optimise the cost-effective culture medium for EPS production in *L. rhamnosus* by modifying single- and multi-factor-at-a-time (i.e. type of carbon and nitrogen sources) through statistical modelling [254]. Meanwhile, RSM such as Central Composite Design (CCD) is a critically acclaimed statistical design that has been used to analyse and evaluate the growth kinetic parameters for the increased EPS production of *L. plantarum* [218]. In brief, the use of economical modelling and statistical designs permit the selection of crucial formulations that influence the production of lactobacilli SAMs that could ultimately lower the overall cost of SAMs production. Besides, the recovery of SAMs from *Lactobacillus* could be improved by using genetically engineered SAMs-producing *Lactobacillus* strain. For instance, Li et al. [258] have shown that the NADH metabolic pathway needed for EPS production can be re-routed to increase the amount of *L. casei* LC2W EPS by 46%. However, the usage of genetically engineered lactobacilli may engender safety concerns among public. The potential risk and safety issue can be addressed by careful experimental inspection before the administration [259]. While a plethora of studies have been carried out in vitro, in vivo testing of *Lactobacillus* SAMs effectiveness to prevent vaginal infection remains understudied. Nonetheless, allogeneic immune response makes the reconstitution of healthy vaginal microbiota to a *Lactobacillus*-dominated microenvironment challenging. Thus, a personalized approach in treating vaginal infections is required to proclaim the beneficial effect of *Lactobacillus* [260]. Besides, high recurrent rate of vaginal infections often suggests the futility of antimicrobial drugs in long-term treatment. With the use of lactobacilli and potentially its derivatives (i.e. SAMs), restoration of a balanced vaginal microbiota could be achieved. Future investigations should focus on creating an economically-feasible approach for large-scale generation of *Lactobacillus* SAMs in order to solve the production bottleneck and to be used as the potential treatment for human vaginal infections.

## Conclusions

The presence of vaginal microbiota and mycobiota in human vagina shape the healthy and diseased state of vaginal ecosystem. Over the past decade, vaginal microbiome profiling has been extensively studied. Numerous studies reported that healthy vaginal CSTs are usually dominated by LAB (i.e. *Lactobacillus*), low diversity of anaerobic bacteria, and a balanced vaginal immune system (e.g. pro-inflammatory and anti-inflammatory cytokines). The host predisposing and genetic factors have been proven to alter vaginal microbial composition. Thus, disrupted vaginal ecosystem often results in diseased state of CST and symptomatic vaginitis. Meanwhile, *Lactobacillus* have shown to possess potential health benefits in immunomodulation and restoration of healthy microflora in gut and vagina. Despite rare *Lactobacillus* bacteraemia reported in immunocompromised patients, their beneficial effects in reducing recurrence rate of vaginal infection and preventing vaginally-acquired infections are well-founded. Therefore, the development of other potential treatments from probiotics should be invested to position the promising benefits of probiotics in immunocompromised patients.

The utilisation of *Lactobacillus* as prophylaxis appeared to be a long-term beneficial approach. As discussed in this review, lactobacilli derivatives (i.e. SAMs) could be utilised as a prevention for vaginal infection via restoration of indigenous microbiota and their anti-biofilm capability. The successful restoration of *Lactobacillus*-dominated composition in BV patients was reported with lower recurrence rate along with the significant decrease in BV-related bacteria such as *Gardnerella*, *Prevotella*, *Megasphaera*, *Coriobacteriaceae*, and *Atopobium* [261]. The beneficial effects of lactobacilli SAMs which act against vaginal pathogens include anti-biofilm, antioxidant, antiviral, pathogen-inhibition, and immunomodulation are proposed to directly involved in the interaction between human host and vaginal microbiota. Considering the ability of *Lactobacillus* SAMs to significantly inhibit the in vitro growth of vaginal pathogens, further studies should be directed on their mechanisms in in vivo model. This will be a valuable tool to facilitate the understanding of role of lactobacilli and derivatives in modulating mucosal barrier of vagina against invading pathogens. The new evidence in the understanding of potential lactobacilli SAMs and their respective mechanistic knowledge will greatly promote the development of prebiotics and antimicrobial agents, aiming on the prevention and treatment of vaginal diseases such as BV, STIs and VVC.

## Data Availability

Not applicable.

## Abbreviations

AV: Aerobic vaginitis
BS: Biosurfactant
BV: Bacterial vaginosis
BVAB: Bacterial vaginosis-associated bacteria
CKD: Chronic kidney disease
COX-2: Cyclooxygenase-2
CST(s): Community state type(s)
CT: Chlamydia trachomatis
ENA-78: Interleukin-5
EPS: Exopolysaccharides
FMT: Faecal microbiota transplantation
GlcNAc: *N*-acetyl-glucosamine
GRAS: Generally recognised as safe
HIV: Human immunodeficiency virus
HMP: Human Microbiome Project
HPV: Human papillomavirus
HSV-2: Herpes simplex virus-2
iHMP: Integrative HMP
IL-12: Interleukin-12
LAB: Lactic acid bacteria
LPS: Lipopolysaccharides
LTA: Lipoteichoic acid
MDA: Malondialdehyde
MRS: de Man, Rogosa and Sharpe
MUBs: Mucin-binding proteins
MurNAc: *N*-acetylmuramic acid
NIH: National Institutes of Health
NO: Nitric oxide
NOD2: Nucleotide-binding oligomerization domain 2
OTUs: Operational taxonomic units
PDMS: Polydimethyl siloxane
PG: Peptidoglycan
PRRs: Pattern recognition receptors
RVVC: Recurrent VVC
SAPs: Secreted aspartyl proteinases
SAMs: Surface-active molecules
SLAPs: Surface layer associated proteins
SOD: Superoxide dismutase
STIs: Sexually transmitted infections
TA: Teichoic acid
TLR2: Toll-like receptor 2
TNF-α: Tumour necrosis factor-α
TSS: Toxic shock syndrome
TSST-1: TSS toxin
UTIs: Urinary tract infections
VMT: Vaginal microbiota transplantation
VVC: Vulvovaginal candidiasis
